# Videos posted on the internet provide evidence for joint rushing in naturalistic social interactions

**DOI:** 10.1038/s41598-023-37247-1

**Published:** 2023-06-30

**Authors:** Thomas Wolf, Tamás Novák, Günther Knoblich

**Affiliations:** 1 Central European University, Vienna, Austria; 2 University of Bristol, Bristol, UK

**Keywords:** Psychology, Human behaviour

## Abstract

When people engage in rhythmic joint actions, they unintentionally increase their tempo. However, this phenomenon of joint rushing has so far been investigated only under very specific and somewhat artificial conditions. Therefore, it remains unclear whether joint rushing generalizes to other instances of rhythmic joint action. In this study our aim was to investigate whether joint rushing can also be observed in a wider range of naturalistic rhythmic social interactions. To achieve this, we retrieved videos of a wide range of rhythmic interactions from an online video-sharing platform. The data suggest that joint rushing indeed can also be observed in more naturalistic social interactions. Furthermore, we provide evidence that group size matters for how tempo unfolds in social interactions with larger groups showing a stronger tempo increase than smaller groups. Comparing the data from naturalistic interactions with data collected in a lab study further showed that unintended tempo changes in social interactions are reduced in naturalistic interactions compared to interactions in a lab context. It is an open question which factors led to this reduction. One possibility is that humans might have come up with strategies to reduce the effects of joint rushing.

## Introduction

When humans engage in joint actions, they coordinate their actions in time and space to bring about change in the environment^1,2^. Many of these joint actions, such as joint music-making, conversation, and many forms of coordinated manual labor, unfold with an inherent rhythmicity^3^. It has been shown that when people engage in rhythmic interactions, they tend to increase the tempo of the interaction unintentionally^4–6^. Most recently, Wolf & Knoblich^6^ have shown that musicians are just as affected by joint rushing as non-musicians.

However, joint rushing has so far been investigated only in the context of synchronized, isochronous tapping that was cued with a metronome set to a target tempo. As this is a very specific and somewhat artificial setting, it remains unclear whether joint rushing generalizes to other instances of rhythmic joint action. Joint music-making, for example, rarely starts with a metronome, and the rhythms are usually more intricate than only isochronous, i.e., regular beats, without any additional rhythmic events. Therefore, in this study our aim was to investigate whether joint rushing can be observed in a wide range of rhythmic social interactions varying in tempo, rhythmicity, and joint action type. To achieve this, we retrieved videos of naturalistic interactions from an online video-sharing platform (YouTube) and selected videos showing rhythmic actions performed in groups of various sizes as well as videos showing rhythmic actions performed alone, as a baseline.

In addition to testing whether the evidence for joint rushing could be extended to naturalistic rhythmic interactions, we also aimed to further investigate the effect of group size on joint rushing. In particular, the model presented by Wolf et al.^5^ to explain joint rushing suggests that group size should be positively correlated with the amount of rushing. In this model human error correction mechanisms that correct for timing errors during sensorimotor synchronization^7^ are combined with a phase advancement mechanism^8–10^ to explain joint rushing. This phase advancement mechanism, as originally proposed in the literature on chorusing insects, triggers a period shortening when taps of others are perceived during a certain time window before one’s own tap. The fact that this mechanism is biased towards a period shortening sets it apart from human correction mechanisms that correct both by shortening or expanding periods^7^. This is the reason why it has been considered as a potential mechanism at play in joint rushing^5^. The relevant time window is also referred to as one’s sensitive window^5^. Because larger groups produce more taps, when performing with a larger group the likelihood is higher that a tap falls into one’s sensitive window and triggers the phase advancement mechanism. In a larger group it is also more likely that a tap falls into the first part of the sensitive window thereby triggering a particularly strong response. Thomson et al.^11^ provide evidence for a systematic relationship between group size and joint rushing in a related task in 31 groups with group sizes ranging from 7 to 220 group members. Larger groups showed a steeper tempo increase than smaller groups.

In this study, we focused on groups ranging from 2 to 20 jointly performing individuals. This range includes group sizes that are common in ensembles for chamber music such as duets, trios, quartets, octets, and other types of chamber ensembles^12^. Finding a difference in joint rushing between smaller and larger groups in our sample would be in line with Thomson et al.’s findings^11^ and corroborate the model of joint rushing proposed by Wolf et al.^5^.

Furthermore, we investigated whether joint rushing in naturalistic settings is reduced compared to joint rushing in lab studies. Wolf & Knoblich^6^ found that in a lab study musicians showed joint rushing to the same extent as non-musicians. As a musician one would hope that in naturalistic settings, as in ensemble music making, joint rushing is less severe and the proper tempo for a piece can be maintained if that is required for a successful performance. The lack of a target tempo given by a metronome at the beginning of an interaction and the involvement of more intricate rhythmic patterns, could reduce joint rushing in naturalistic settings. To quantify such effects, we compared tempo changes derived from videos of naturalistic interactions to tempo changes obtained in isochronous beat-based tapping studies obtained in the laboratory. If joint rushing is less pronounced in naturalistic settings, we could conclude that some characteristics of naturalistic rhythmic interactions help to reduce joint rushing. This could be a first indication that joint rushing can be mitigated. For the laboratory data, with which to compare the real-world data, we used the lab data published in Wolf & Knoblich^6^ as this lab data includes both musicians and non-musicians, something that we can also expect from our online data and the length of the interactions in this lab study is identical to the length of our video snippets, i.e., 60 s.

In the current study, we therefore aimed to address three main questions. First, we asked whether there is evidence for joint rushing in more naturalistic social interactions. Second, does group size influence joint rushing? And third, how does joint rushing in real-world interactions compare to joint rushing in lab experiments?

In addition to our main hypotheses, there were two further hypotheses included in the pre-registration which, in light of research conducted in the interim, we no longer consider to be well-motivated. The analyses pertaining to these two hypotheses, together with references to relevant research, are included in the supplementary material (see SM4).

## Methods

### Video sampling

We aimed to collect a sample of videos showing rhythmic activities performed by either one person (solo condition) or by a group of people (group condition). During the selection process we used the following four selection criteria. (1) Videos should show rhythmic activity with a minimum duration of one minute. When we started this project, we had no data available that provided us with information about how long it would take for joint rushing to produce a measurable tempo increase. The limit of one minute was chosen for practical reasons, because we expected that most videos of uninterrupted rhythmic interactions would be relatively short. In the meantime, lab studies have provided evidence that joint rushing can be measured within a time span of one minute^5,6^. Note that this would also exclude videos that did not show rhythmic activity at all. (2) Videos in the group condition should show group sizes ranging from two to twenty people. This range of group sizes corresponds to typical ensemble sizes in the Western classical music tradition and enabled us to compare joint rushing in small and larger groups. This criterion was neglected for videos in the solo condition. (3) No external timekeeper, such as a metronome or the playback of pre-recorded music present in the video to restrict the sample to unaided human tempo keeping behavior. (4) No video cuts or other evidence indicating that the audio and the video were recorded separately. Separately recorded audio implies the possibility that a click-track or metronome was used during the audio recording (see 3). The aim was to collect as many videos as possible in the time frame of the project. Video scraping, the extraction of video from a webpage, was done with the help of the official YouTube Data Application-Programming Interface (API), which provides simple rules and functions to interface with YouTube’s data. We requested a total of 300 videos through this API and filtered them according to the criteria described above. This resulted in a sample of 34 videos in the group condition that all adhered to our four selection criteria and 11 videos in the solo condition that adhered to criteria 1, 3 and 4.

To collect videos that adhere to the selection criteria we developed a repeated, semi-automatic sampling process that included the three steps keyword-based/relation-based search, automatic filtering, and manual classification. We used automatic filtering where possible, e.g., to select for videos that are longer than one minute (selection criterion 1), but then also used manual classification to check for example whether the rhythmic interactions were uninterrupted and lasted for at least one minute (also selection criterion 1).

At the time of the video sampling there was no evidence yet on whether for joint rushing to occur the participants in the rhythmic activity should have visual access to each other’s movements. For this reason, we decided to start searching for videos with rhythmic activity that is easily visible, such as body percussion activity. Hence, we started the first round of a keyword-based search with the keyword “body percussion” to ensure rhythmic activity that includes both visual and auditory action effects. We reasoned that this approach would allow us to identify a large enough sample of relevant videos to identify further keywords in the second round. The YouTube API allowed us to download additional information besides the video IDs, such as video title, video duration, number of views, category, uploader and channel name.

After the first implementation of our three-step sampling process we had data about which categories, channels, uploaders and keywords were more likely to yield videos that adhere to our selection criteria. For the second round, this allowed us to search for videos by relationship to usable videos from sampling round one (relation-based search) and to identify further promising keywords such as “pen tapping”, “energizers” and “bring me water Sylvie” (keyword-based search). “Energizers” are short game-like songs that usually involve body percussion elements. “Bring me water Sylvie” is a song that is typically accompanied by a body percussion routine. A table in the supplementary material (see SM1) lists all 45 videos we ended up with and whether they were added to the sample by way of keyword search, in which case the specific keyword is given, or whether they were related to usable videos from previous rounds.

### Video coding

Videos were then coded by two of the authors for number of people involved in the rhythmic action, movement similarity, and task complexity. Also, time stamps were added for the beginning and the end of uninterrupted rhythmic actions.

### Computerized and manual tempo detection

Each video was cut to start at the beginning of the rhythmic action and to end after one minute. The audio was extracted from all resulting video snippets. For the computerized tempo detection video snippets were further cut into pieces of 10 s each. First, we used a commercially available tempo detection software (beaTunes, v5.0.6) to extract the tempo. However, the relatively low signal-to-noise ratio in our video snippets (most of which were amateur recordings) led to unreliably fluctuating tempo values.

Therefore, to establish the tempo, we invited expert musicians to tap along with the beat of the recorded rhythmic actions. We chose to invite exactly three musicians to deal with the possibility that some videos have more than one salient metric level, which could lead to an ambiguity in tempo perception^13^. With the odd number three, we expected at least two of the tappers to converge on the most salient metric level. We were able to recruit two classical drummers (10 years of private lessons, currently playing 8 h per week, 2 years of teaching, 43 years of ensemble playing, as well as 22 years of private lessons, currently playing 35 h per week, 14 years of teaching, 17 years of ensemble experience) and one classical guitarist (15 years of private lessons, currently playing 5 h per week, 25 years of teaching, 5 years of ensemble experience) who all studied their respective instruments and have pursued a career as professional musicians. These expert musicians gave their informed consent and received a monetary compensation. All experiments in this study were conducted in accordance with the Declaration of Helsinki, approved by the Psychological Research Ethics Board (PREBO) at CEU, Vienna, Austria and pre-registered (https://doi.org/10.17605/OSF.IO/XYAEW).

We invited each expert musician to tap along with the audio track of each video twice and to indicate in which of the two takes they felt they better matched the beat of the audio track. In all but one case this was the second instance. Taps were recorded with a custom Max MSP patch (Max version 8.5.0) run on an intel-based MacBook Air and a Sensel Morph as input device. To extract tempo estimates in terms of inter-beat-intervals (IBIs) from expert musicians’ inter-tap-intervals (ITIs), we aggregated and averaged them into time bins of 10 s each. We removed ITIs that were smaller than the mean minus two times the standard deviation and larger than the mean plus two times the standard deviation. This was calculated per video, tapper and time bin and done to remove occasional double triggers or omissions in the detection of taps by the Sensel Morph. This procedure resulted in the removal of 4.26% of all ITIs.

As expected, some videos had multiple salient metric levels^13^ and therefore the three tappers perceived the beat to be on different metric levels. In these cases, there were always two tappers who heard the beat on the same metric level. To account for these subjective differences in determining the metric level of the beat and to get tight temporal measures, we used the two of the three ratings that were closest to each other for each video and discarded the third. To determine the two closest ratings, we summed the absolute differences for all three possible pairings for each time bin and chose the pairing with the lowest sum of absolute differences. To assess the interrater reliability, we calculated the intraclass correlation coefficient (ICC), which ranges from 0 (low reliability) to 1 (perfect reliability). This showed that there was excellent agreement between rater 1 and rater 2 (ICC = 0.989, p < 0.001), between rater 1 and rater 3 (ICC = 0.999, p < 0.001), as well as between rater 2 and rater 3 (ICC = 0.999, p < 0.001). When we calculated individual ICCs for each video separately, the average was 0.844 (considered to be good reliability) with a standard deviation of 0.17. A one-sampled Wilcoxon test, which we used, because these ICCs were not distributed normally, showed that they were significantly higher than the lower limit of good reliability (lower limit = 0.75): *V*_*Wilcoxon*_ = 824.00, *p* < 0.001 (for a table of the individual ICCs and corresponding p-values for each video see SM5 in the supplementary material).

### Analysis

Since we expressed tempo in terms of IBIs, lower values of IBIs indicate faster tempi. To make tempo changes comparable across videos with different initial tempi, we normalized IBIs in such a way that the IBI average in all time bins is divided by IBI average in the initial time bin. This means that the initial time bin always has a normalized tempo of 1. While in time bins 2 to 6 a normalized tempo smaller than 1 stands for a tempo faster than the initial tempo and a normalized tempo larger than 1 stands for a tempo slower than the initial tempo. To also compare the absolute tempo of videos we use the non-normalized IBI values which revealed the same critical pattern of joint rushing (see SM2 and SM3 in the supplementary material).

To analyze the effect of group sizes, we performed a median split by group size of all videos that feature joint performances. This led to a set of 17 videos that depict “small groups”, i.e., groups with a group size between two and four and a set of 17 videos that depict “large groups”, i.e., groups with a group size between 6 and 20 (see Table 1).

**Table 1:** This table shows 11 solo videos and the distribution of all 34 group videos across group sizes and group types.

	Solo	Small Groups			Large groups									
Group size	1	2	3	4	6	7	8	9	12	13	14	16	17	20
No. of videos	11	4	4	9	1	1	3	2	2	2	2	1	1	2
Sum	11	17			17									

The lab data for the comparison between data from YouTube and data gathered in the lab was taken from Wolf & Knoblich^6^. Since Wolf & Knoblich found no significant difference between joint rushing in musicians and non-musicians and the people depicted in the YouTube videos appear to include both musicians and non-musicians the lab data from 24 musicians and 24 non-musicians was combined.

All ANOVA results reported in this paper were computed with the ‘ez’ package (version 4.4.0) for R (version 4.2.2)^14,15^. For all results that include factors with more than two levels we report the Greenhouse–Geisser corrected values provided by the function ezANOVA().

## Results

### Comparing solo and group videos

**Figure 1:**
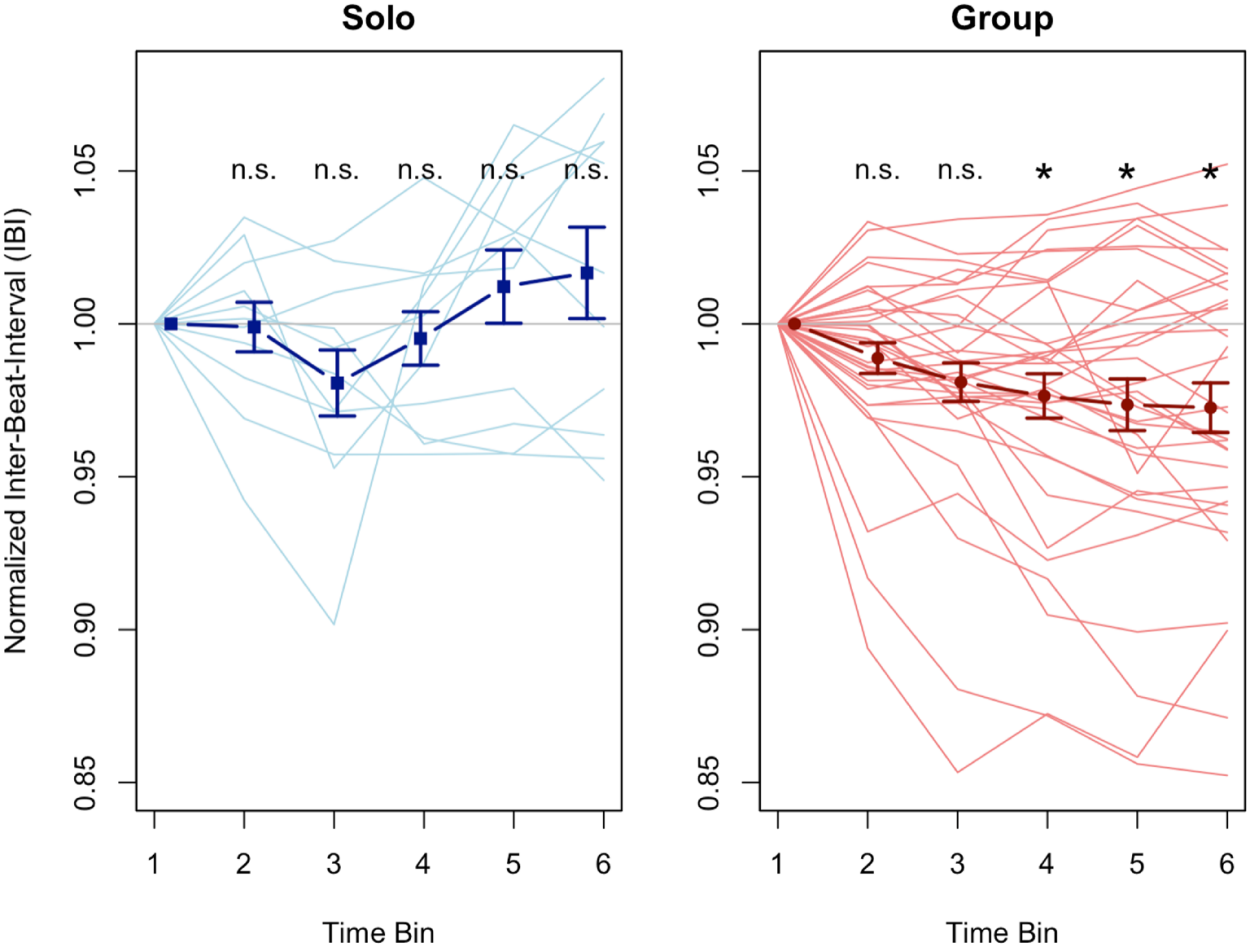
The plots here show the normalized Inter-Beat-Intervals (IBI) in Solo and Group videos. Lower IBIs mean faster tempo. Values below 1 mean the tempo was faster than the initial tempo and values above 1 mean the tempo was slower than the initial tempo. Each time bin is 10 s long. Light blue and light red lines show the tempo development of individual solo and individual group videos respectively. The dark blue and the dark red line show group averages for solo and group videos respectively. Error bars show standard error. Significances show the Bonferroni-corrected values of one-sample t-tests (IBI in a given bin tested against 1, i.e., against the starting tempo), corrected for 10 tests. Only in group videos do we see a tempo increase that leads to a tempo which is significantly faster than the starting tempo.

In order to address the question of whether there is evidence for joint rushing in naturalistic data, we entered Normalized Inter-Beat-Intervals in a 2 × 6 ANOVA with the between factor Setting (Solo or Group) and the within factor Time Bin (1 to 6), see Fig. 1. The interaction between the two factors Setting and Time Bin was significant (*F*(5, 215) = 5.624, *p* = 0.003, η^2^ = 0.041). The main effect for Time Bin was also significant (*F*(5, 215) = 4.817, *p* = 0.007, η^2^ = 0.035). There was no significant main effect of Setting (*F*(1, 43) = 3.100, *p* = 0.085).

To interpret the significant interaction, we computed pairwise comparisons between normalized IBIs in Solo and Group videos for the time bins 2 and 6 (time bin 1 has the initial tempo which through the normalization process had the value 1 for each video). With a Bonferroni-corrected threshold of significance of alpha = 0.025 the results showed a significant difference between Solo and Group in time bin 6 (*t*(16.351) = − 2.594, *p* = 0.019, *d* = 0.921, Solo (*M* = 1.017, *SD* = 0.050), Group (*M* = 0.973, *SD* = 0.047)), but no significant difference in time bin 2 (*t*(18.311) = − 1.066, *p* = 0.300, Solo (*M* = 0.999, *SD* = 0.027), Group (*M* = 0.989, *SD* = 0.029)). This means that the tempo in Solo videos did not significantly differ from the tempo in Group videos after 20 s, but it did differ after 60 s.

To check for further evidence of rushing, we computed pairwise comparisons between the tempo in time bin 2–6 and the initial tempo, i.e., the tempo in time bin 1. Since the tempo was normalized such that the initial tempo is 1, we in fact computed one-sampled t-tests for the tempo in time bins 2–6 against the value 1. With a Bonferroni-corrected threshold of significance of alpha = 0.005 the results showed significantly lower normalized IBIs than 1, i.e., a significant tempo increase, in the group setting in time bin 4 (*t*(33) = − 3.247, *p* = 0.003, *d* = 0.557, *M* = 0.976, *SD* = 0.042), time bin 5 (*t*(33) = − 3.133, *p* = 0.004, *d* = 0.537, *M* = 0.974, *SD* = 0.049) and time bin 6 (*t*(33) = − 3.376, *p* = 0.002, *d* = 0.579, *M* = 0.973, *SD* = 0.047), but no significant difference in time bin 2 (*t*(33) = − 2.232, *p* = 0.033, *M* = 0.989, *SD* = 0.029) and time bin 3 (*t*(33) = − 3.029, *p* = 0.005, *M* = 0.981, *SD* = 0.037). In the solo setting none of the time bins showed a significant difference from the initial tempo—time bin 2 (*t*(10) = − 0.128, *p* = 0.901, *M* = 0.999, *SD* = 0.027), time bin 3 (*t*(10) = − 1.791, *p* = 0.104, *M* = 0.981, *SD* = 0.036), time bin 4 (*t*(10) = − 0.545, *p* = 0.598, *M* = 0.995, *SD* = 0.029), time bin 5 (*t*(10) = 1.017, *p* = 0.333, *M* = 1.012, *SD* = 0.040), time bin 6 (*t*(10) = 1.117, *p* = 0.290, *M* = 1.017, *SD* = 0.050). This means that there was a significant tempo increase in the group videos but not in the solo videos (see Fig. 1), or in other words, rushing only took place in group videos, a clear indication of joint rushing.

### Comparing small and large groups

**Figure 2:**
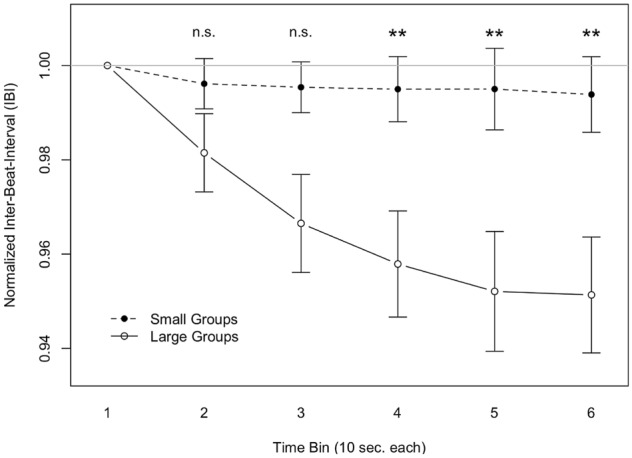
The plot shows the normalized Inter-Beat-Intervals (IBI) in Solo and Group videos. Lower IBIs mean faster tempo. Values below 1 mean the tempo was faster than the initial tempo. Each time bin is 10 s long. The dashed line shows the average for Small Groups (17 small groups of 2–4 people) and the solid line shows the average for Large Groups (17 large groups of 6–20 people). Error bars show standard error. Significances show the Bonferroni-corrected values, corrected for 5 tests. A significant interaction between the factors Group Type and Time Bin and post-hoc tests show that the steeper tempo increase in large groups leads to significant tempo differences between small and large groups in time bins 4, 5 and 6.

The next analysis addressed the question of whether group size affects joint rushing. For this analysis, we only used group videos and we entered their Normalized IBI in a 2 × 6 ANOVA with the between factor Group Type (Small or Large) and the within factor Time Bin (1 to 6), see Fig. 2. The interaction between the two factors Group Type and Time Bin was significant (*F*(5, 160) = 6.419, *p* = 0.002, η^2^ = 0.051). The main effect of Group Type was also significant (*F*(1, 32) = 7.292, *p* = 0.011, η^2^ = 0.143). There was also a significant main effect of Time Bin (*F*(5, 160) = 9.769, *p* < 0.001, η^2^ = 0.076).

To interpret the significant interaction, we computed pairwise comparisons between the tempo in small groups and the tempo in large groups for the time bins 2–6. With a Bonferroni-corrected threshold of significance of alpha = 0.01 the results showed significant differences between the two group types in time bin 4 (*t*(26.577) = 2.809, *p* = 0.009, *d* = 0.964, small groups (*M* = 0.995, *SD* = 0.028), large groups (*M* = 0.958, *SD* = 0.046)), time bin 5 (*t*(28.216) = 2.791, *p* = 0.009, *d* = 0.957, small groups (*M* = 0.995, *SD* = 0.036), large groups (*M* = 0.952, *SD* = 0.052)) and time bin 6 (*t*(27.528) = 2.897, *p* = 0.007, *d* = 0.994, small groups (*M* = 0.994, *SD* = 0.033), large groups (M = 0.951, SD = 0.051)), but no significant difference in time bin 2 (*t*(27.305) = 1.487, *p* = 0.148, small groups (*M* = 0.996, *SD* = 0.022), large groups (*M* = 0.981, *SD* = 0.034)) and time bin 3 (*t*(24.04) = 2.466, *p* = 0.021, small groups (*M* = 0.995, *SD* = 0.022), large groups (*M* = 0.967, *SD* = 0.043)). This means that there was significantly more increase in normalized tempo over time bins in large groups than in small groups (see Fig. 2). In fact, for small groups there was no evidence for a tempo increase. A one sampled t-test that compared the normalized IBIs in time bin 6 against the initial tempo, i.e., against one, yielded no significant difference, t(16) = − 0.767, p = 0.454, M = 0.994, SD = 0.033.

### Comparing YouTube data to lab data

**Figure 3:**
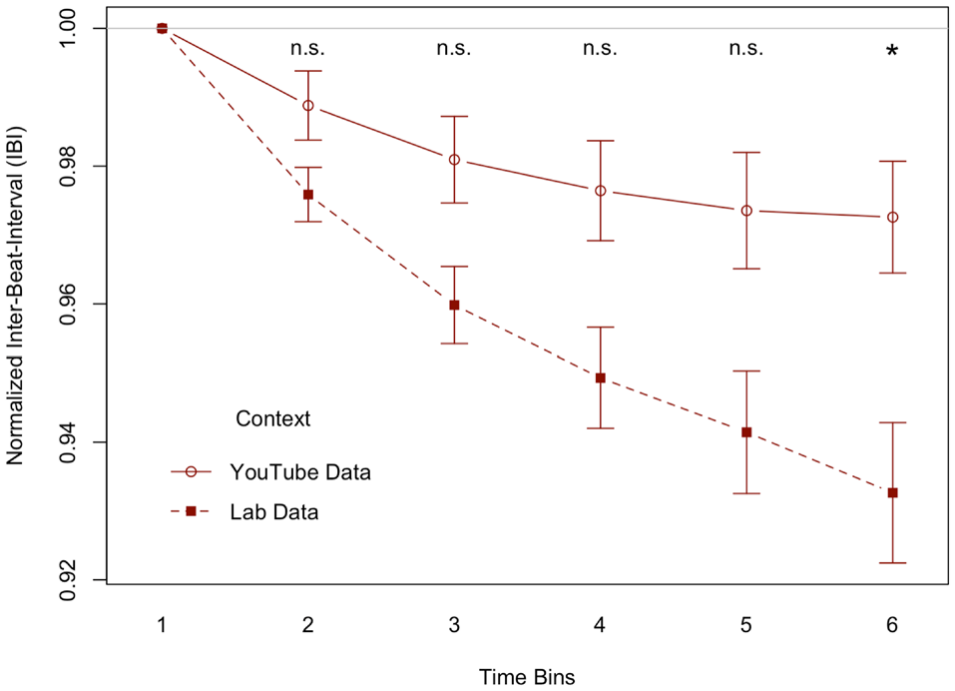
The plot shows the normalized Inter-Beat-Intervals (IBI) for group videos in the YouTube data and for lab data gathered in a joint setting. Lower IBIs mean faster tempo. Values below 1 mean the tempo was faster than the initial tempo. Each time bin is 10 s long. The solid line shows average normalized IBIs for YouTube data and the dashed line shows the average normalized IBIs for Lab data. Error bars show standard error. Significances show the Bonferroni-corrected values, corrected for 5 tests. The significant interaction between the factors Context and Time Bin and the post-hoc tests show that by time bin 6 the steeper tempo increase in the Lab Data leads to a significantly faster tempo in the Lab data than in the YouTube data.

This analysis was computed to investigate differences in joint rushing between data from real-world interactions and lab interactions. For this analysis, we only used group videos from the current study to compare it to data from the Joint condition in the lab experiments described in Wolf & Knoblich^6^. This allowed us to compare the data from 34 group videos with the Joint condition data from 48 lab participants. For both data sets we calculated Normalized IBI in the same way. We entered this data in a 2 × 6 ANOVA with the between factor Context (YouTube or Lab) and the within factor Time Bin (1 to 6), see Fig. 3. The interaction between the two factors Context and Time Bin was significant (*F*(4, 320) = 6.179, *p* = 0.009, η^2^ = 0.009). The main effect of Context was also significant (*F*(1, 80) = 6.936, *p* = 0.014, η^2^ = 0.071). There was also a significant main effect of Time Bin (*F*(4, 320) = 37.531, *p* < 0.001, η^2^ = 0.053).

To interpret the significant interaction, we computed pairwise comparisons between the tempo in the YouTube context and the tempo in the Lab context for the time bins 2–6. With a Bonferroni-corrected threshold of significance of alpha = 0.01 the results showed a significant difference between the two contexts in time bin 6 (*t*(79.806) = 3.067, *p* = 0.003, *d* = 0.644, YouTube context (*M* = 0.973, *SD* = 0.047), Lab context (*M* = 0.933, *SD* = 0.071)). The differences were not significant for the other time bins; time bin 2 (*t*(68.030) = 2.027, *p* = 0.047, YouTube context (*M* = 0.989, *SD* = 0.029), Lab context (*M* = 0.976, *SD* = 0.027)), time bin 3 (*t*(73.566) = 2.507, *p* = 0.014, YouTube context (*M* = 0.981, *SD* = 0.037), Lab context (*M* = 0.960, *SD* = 0.039)), time bin 4 (*t*(77.758) = 2.632, *p* = 0.010, YouTube context (*M* = 0.976, *SD* = 0.042), Lab context (*M* = 0.949, *SD* = 0.051)) and time bin 5 (*t*(78.757) = 2.619, *p* = 0.011, YouTube context (*M* = 0.974, *SD* = 0.049), Lab context (*M* = 0.941, *SD* = 0.062)). This means that there was significantly more increase in normalized tempo over time bins in the Lab context than in the YouTube context (see Fig. 3).

## Discussion

The aim of this study was to investigate joint rushing in naturalistic rhythmic interactions uploaded to the video sharing platform YouTube. Our first question was whether there is evidence for joint rushing in these real-world joint actions. Our results showed that there is a systematic tempo increase in group videos, while there was no significant tempo change in solo videos. This provides evidence that joint rushing is not limited to rhythmic interactions in lab studies with a target tempo and synchronization of isochronous beats but does also occur in real-world interactions.

Our second question was whether group size influences the effect of joint rushing, or to be more specific whether larger groups tend to exhibit stronger joint rushing than smaller groups. Our results showed stronger joint rushing for larger groups (seven to twenty people) than for smaller groups (two to six people). This is in line with findings by Thomson et al.^11^ who also found a correlation between group size and tempo increase in a hand clapping task. The bulk of the data, however, came from groups with more than twenty people. Our dataset of 34 videos of groups with two to twenty people therefore extends Thomson et al.’s findings to groups under 20 people. These findings are in line with the joint rushing model proposed by Wolf et al.^5^, in which phase advancement^8–10^ plays an important role. According to this model more people engaging in rhythmic activity should lead to more signals that have the potential to fall into each other’s sensitive windows. This in turn should lead to a higher rate of triggered phase advancement events.

Nonetheless, the current group size finding and Thomson et al.’s findings need to be reconciled with data by Wolf et al.^5^ who found no evidence for a difference in rushing between groups of two and groups of three in a lab study. One obvious difference lies in the group sizes that are being compared. While Thomson et al. cover groups from 7 to 220 and in the current study we compare small groups (2–4) to larger groups (6–20), Wolf et al.^5^ compared groups of two with groups of three. More data points from interactions with groups in the range from two to five members would be needed to assess whether there is a linear relationship between group size and joint rushing already in these types of groups.

Our third question was about how joint rushing in real-world interactions compare to joint rushing data collected in a lab study. The comparison showed that joint rushing was significantly reduced in the real-world interactions as compared to joint rushing in the lab study. While the average Inter-Beat-Interval was reduced by 2.74% after 60 s in the sample of YouTube videos, the lab interactions saw a reduction of 6.74% after 60 s. In small groups (two to four people) the nominal tempo change was in fact not significant at all, whereas the lab study showed a significant tempo change in groups of two.

This difference in joint rushing is unlikely to be related to the possibility that people who choose to upload or agree to have their interactions uploaded to YouTube might have had significant amounts of musical training. Wolf & Knoblich^6^ found no difference in joint rushing between a sample of non-musicians and a sample of musicians with significant amount of musical training. However, there were several important differences between the interactions shown in the videos and the lab interactions that could account for the difference in joint rushing.

First, participants in the lab study were always cued with a target tempo of 120 bpm, which corresponds to an Inter-Beat-Interval of 500 ms, whereas the interactions in the videos started with different tempi. However, the average starting tempo was not significantly different from an IBI of 500 ms (see SM2 in the supplementary material for absolute IBIs).

A second difference between the social interactions on video and the social interactions in the lab is their rhythmic structure. While all lab interactions consisted solely of isochronous beats not a single video interaction did. All video interactions in our sample were based on rhythmic behavior that included beat subdivisions. The so-called subdivision benefit is known to reduce variability of asynchronies^16^. This could be important, because all proposed models of joint rushing predict a positive relationship between variability and joint rushing where more variability should lead to stronger rushing. Here, beat subdivisions might reduce the variability and thereby also joint rushing^5,11,17,18^. However, in another study Repp^19^ concluded that subdivisions lead to a subjective slowing of the beat. Interaction partners might feel the need to counteract this subjective slowing by speeding up. This in turn might neutralize the rushing-reducing effect of the subdivision benefit. Further experiments are needed to assess whether and how beat subdivision affects the tempo in social interactions.

A third difference is that in the lab study participants used one finger to tap throughout the whole experiment, whereas in all group videos at least two effectors were used at some point. This could be important, as the multiple effector advantage^20–22^ also reduces variability. In fact, variability is reduced even further when three effectors are used concurrently^23^, as it was the case in 27 of the 34 group videos in our sample. As discussed above, models of joint rushing would predict a reduction in rushing to follow from a reduction in motor variability.

A fourth difference between the lab studies and the videos was that in several videos the rhythmic behavior included rhythmic vocalizations such as singing or speaking along. On the one hand, speaking concurrently with tapping has been found to increase the variability of tapping^24^, which should theoretically increase joint rushing. On the other hand, concurrent speaking can also lead to a reduction in the rate at which children of various ages are tapping when asked to tap as fast as possible^24,25^. However, it is unclear whether this also translates to adults tapping at a much slower rate than their performance limit. Another indication that concurrent vocalization might help to prevent joint rushing comes from the literature on work songs. It seems that one of the many proposed functions of work songs^26^ has to do with keeping a certain “measured tempo” (p.121) and to “maintain the proper pace” (p. 135) when “maintaining the proper speed is critical to the success of the work”^27^. When we think of the possible use of work songs as tempo regulation strategies, it could be the case that these strategies work better for smaller groups than for larger groups. Again, more research is needed to investigate the possible effects of concurrent speech on the tempo of rhythmic social interactions.

With regards to collecting data from YouTube for behavioral research we want to briefly discuss the potential issue of consent. As Berger^28^ points out YouTube users who upload videos are required to ensure that consent for being recorded has been obtained and YouTube takes down non-consensual recordings. Hence, users and researchers can assume that consent to be recorded has been obtained for videos that have not been removed. However, it is important to note that this might change as YouTube’s community guidelines and policies are subject to regular updates.

Overall, this study provides evidence for the occurrence of joint rushing in real-world rhythmic interactions, as well as for the influence of group size on joint rushing. The evidence provided also suggests that joint rushing is reduced in naturalistic social interactions compared to interactions in a lab context. These findings are important for temporal coordination in social interactions for several reasons. First, they exemplify how looking into less controlled, but more naturalistic data of social interactions can bring new insights and generate important questions for future research. Second, it is an indication that the implementation of mechanisms underlying interpersonal coordination might lead to different outcomes depending on the size of a group. Most research on joint action, however, is done with dyads. Third, the occurrence of joint rushing in naturalistic interactions means that unintended tempo changes are a real problem for social interactions that require a certain pace or need to be maintained for a long time^26,27^. Fourth, the possibility of mitigating joint rushing opens the door to questions about which individual or joint strategies help to do so. One of the possible candidates discussed above is the addition of beat subdivisions to rhythmic interactions, e.g., by concurrently singing along with the task as in work songs. Are songs, and especially work songs, therefore a tool that evolved to prevent unintended tempo changes in social interactions? This and similar questions could be addressed through controlled experiments, but also to some extent by large scale corpus studies. In order for large scale corpus studies to address these questions, difficulties that we encountered in automatizing the sampling and filtering of videos and in automatically but reliably extracting the tempo need to be overcome.

## Supplementary Information

Supplementary Information.

## Data Availability

The datasets generated and/or analyzed during the current study are available in the OSF repository: https://osf.io/8c7u2/?view_only=04be1189a5dd4d07b8d0e46bcb6e1df5.
