# Supplementary material for: Videos posted on the internet provide evidence for joint rushing in naturalistic social interactions
Source: Sci Rep. 2023 Jun 30;13:10584. doi: 10.1038/s41598-023-37247-1 (PMC10313718; doi:10.1038/s41598-023-37247-1)
Supplement: Supplementary file 1 — Supplementary Information. [file 41598_2023_37247_MOESM1_ESM.docx]

**Supplementary Material for Videos Posted on the Internet Provide Evidence for Joint Rushing in Naturalistic Social Interactions**

**SM1 – Table of all videos in the sample**

In cases where the link is not functional anymore, you might be able to view a snapshot from an earlier time using wayback machine (<https://web.archive.org/>).

| **Video ID** | **Keyword / Relation** | **Number of People** | **Similar Movement** | **Task Difficulty** | **Link** |
| --- | --- | --- | --- | --- | --- |
| -6VD_ZdTzPk | body percussion | 1 | - | 4 | <https://www.youtube.com/watch?v=-6VD_ZdTzPk> |
| 0IOMI5GGP1g | pen tapping | 1 | - | 3 | <https://www.youtube.com/watch?v=0IOMI5GGP1g> |
| 7TTAOuNToGs | body percussion | 1 | - | 4 | <https://www.youtube.com/watch?v=7TTAOuNToGs> |
| EqusX23ZkAk | pen tapping | 1 | - | 4 | <https://www.youtube.com/watch?v=EqusX23ZkAk> |
| f8bkvO1XpzM | body percussion | 1 | - | 5 | <https://www.youtube.com/watch?v=f8bkvO1XpzM> |
| fBeyDR84qKY | body percussion | 1 | - | 4 | <https://www.youtube.com/watch?v=fBeyDR84qKY> |
| GP_aV_SdJLc | pen tapping | 1 | - | 4 | <https://www.youtube.com/watch?v=GP_aV_SdJLc> |
| hhHZ5tAruA4 | body percussion | 1 | - | 3 | <https://www.youtube.com/watch?v=hhHZ5tAruA4> |
| l0XdDKwFe3k | body percussion | 1 | - | 5 | <https://www.youtube.com/watch?v=l0XdDKwFe3k> |
| oFeWJaqqLrQ | body percussion | 1 | - | 5 | <https://www.youtube.com/watch?v=oFeWJaqqLrQ> |
| VVLl0C65NR8 | body percussion | 1 | - | 4 | <https://www.youtube.com/watch?v=VVLl0C65NR8> |
| E1SIF3REWBs | body percussion | 2 | yes | 2 | <https://www.youtube.com/watch?v=E1SIF3REWBs> |
| i0HEqE53WL4 | pen tapping | 2 | no | 3 | <https://www.youtube.com/watch?v=i0HEqE53WL4> |
| pH6HYGjdc48 | body percussion | 2 | yes | 2 | <https://www.youtube.com/watch?v=pH6HYGjdc48> |
| XBuJJ_luB90 | body percussion | 2 | no | 4 | <https://www.youtube.com/watch?v=XBuJJ_luB90> |
| r1ZQbEKiZ8o | related | 3 | no | 3 | <https://www.youtube.com/watch?v=r1ZQbEKiZ8o> |
| u6cKxw0zkfc | bring me water sylvie | 3 | yes | 2 | <https://www.youtube.com/watch?v=u6cKxw0zkfc> |
| u7yVMspnKTc | bring me water sylvie | 3 | yes | 2 | <https://www.youtube.com/watch?v=u7yVMspnKTc> |
| wYcLAC63pGI | body percussion | 3 | no | 4 | <https://www.youtube.com/watch?v=wYcLAC63pGI> |
| 3ikYFd9vTbk | bring me water sylvie | 4 | yes | 3 | <https://www.youtube.com/watch?v=3ikYFd9vTbk> |
| B2UcIaIe2xE | related | 4 | yes | 3 | <https://www.youtube.com/watch?v=B2UcIaIe2xE> |
| eIiYQN3pvt4 | bring me water sylvie | 4 | yes | 3 | <https://www.youtube.com/watch?v=eIiYQN3pvt4> |
| EULpbJQLzSg | related | 4 | no | 5 | <https://www.youtube.com/watch?v=EULpbJQLzSg> |
| FWmoLP7A0MY | body percussion | 4 | no | 4 | <https://www.youtube.com/watch?v=FWmoLP7A0MY> |
| P_POu_za5h0 | bring me water sylvie | 4 | yes | 3 | <https://www.youtube.com/watch?v=P_POu_za5h0> |
| SkHGhGpaW5k | bring me water sylvie | 4 | yes | 3 | <https://www.youtube.com/watch?v=SkHGhGpaW5k> |
| SX_pGYojtxc | body percussion | 4 | yes | 3 | <https://www.youtube.com/watch?v=SX_pGYojtxc> |
| xISEWJrs4iU | bring me water sylvie | 4 | yes | 3 | <https://www.youtube.com/watch?v=xISEWJrs4iU> |
| IwkPtUtNrvw | related | 6 | no | 4 | <https://www.youtube.com/watch?v=IwkPtUtNrvw> |
| uhRAOFx-vLY | body percussion | 7 | yes | 3 | <https://www.youtube.com/watch?v=uhRAOFx-vLY> |
| n0qs4_bY4vg | related | 8 | yes | 3 | <https://www.youtube.com/watch?v=n0qs4_bY4vg> |
| U90mzAvCHGA | bring me water sylvie | 8 | yes | 2 | <https://www.youtube.com/watch?v=U90mzAvCHGA> |
| UY3XxnRtu48 | bring me water sylvie | 8 | yes | 3 | <https://www.youtube.com/watch?v=UY3XxnRtu48> |
| 5i8rG5rKzkY | energizers | 9 | yes | 2 | <https://www.youtube.com/watch?v=5i8rG5rKzkY> |
| LQTol51C4oU | body percussion | 9 | no | 4 | <https://www.youtube.com/watch?v=LQTol51C4oU> |
| 23Xust2zxbM | energizers | 12 | yes | 3 | <https://www.youtube.com/watch?v=23Xust2zxbM> |
| 5AhrTeBVW4M | body percussion | 12 | no | 3 | <https://www.youtube.com/watch?v=5AhrTeBVW4M> |
| LmC6Qyjk8cw | energizers | 13 | yes | 3 | <https://www.youtube.com/watch?v=LmC6Qyjk8cw> |
| wbxidzAdi4c | energizers | 13 | yes | 2 | <https://www.youtube.com/watch?v=wbxidzAdi4c> |
| -3MVWpXrB5o | energizers | 14 | yes | 1 | <https://www.youtube.com/watch?v=-3MVWpXrB5o> |
| 6XDQutQdHBI | energizers | 14 | yes | 2 | <https://www.youtube.com/watch?v=6XDQutQdHBI> |
| 6izJ39ChKpI | body percussion | 16 | no | 4 | <https://www.youtube.com/watch?v=6izJ39ChKpI> |
| pf_xLrhjjfI | body percussion | 17 | no | 3 | <https://www.youtube.com/watch?v=pf_xLrhjjfI> |
| g7Wjl9x4N3U | body percussion | 20 | yes | 1 | <https://www.youtube.com/watch?v=g7Wjl9x4N3U> |
| VZRypNsVBns | related | 20 | no | 3 | <https://www.youtube.com/watch?v=VZRypNsVBns> |

**SM2 – Solo versus Group videos in absolute Inter-Beat-Intervals**

We entered Absolute IBI in a 2 × 6 ANOVA with the between factor Setting (Solo or Group) and the within factor Time Bin (1 to 6).

Interaction*: *F*(5, 215) = 5.478, *p* = .003, η2 = 0.002

Main effect of Time Bin*: *F*(5, 215) = 4.855, *p* = .006, η2 = 0.002

Main effect of Setting: *F*(1, 43) = 0.056, *p* = .814, η2 = 0.001

One-sample t-test of the starting tempo in group videos against an Inter-Beat-Interval of 500 ms: *t*(33) = 1.615, *p* = .116

**
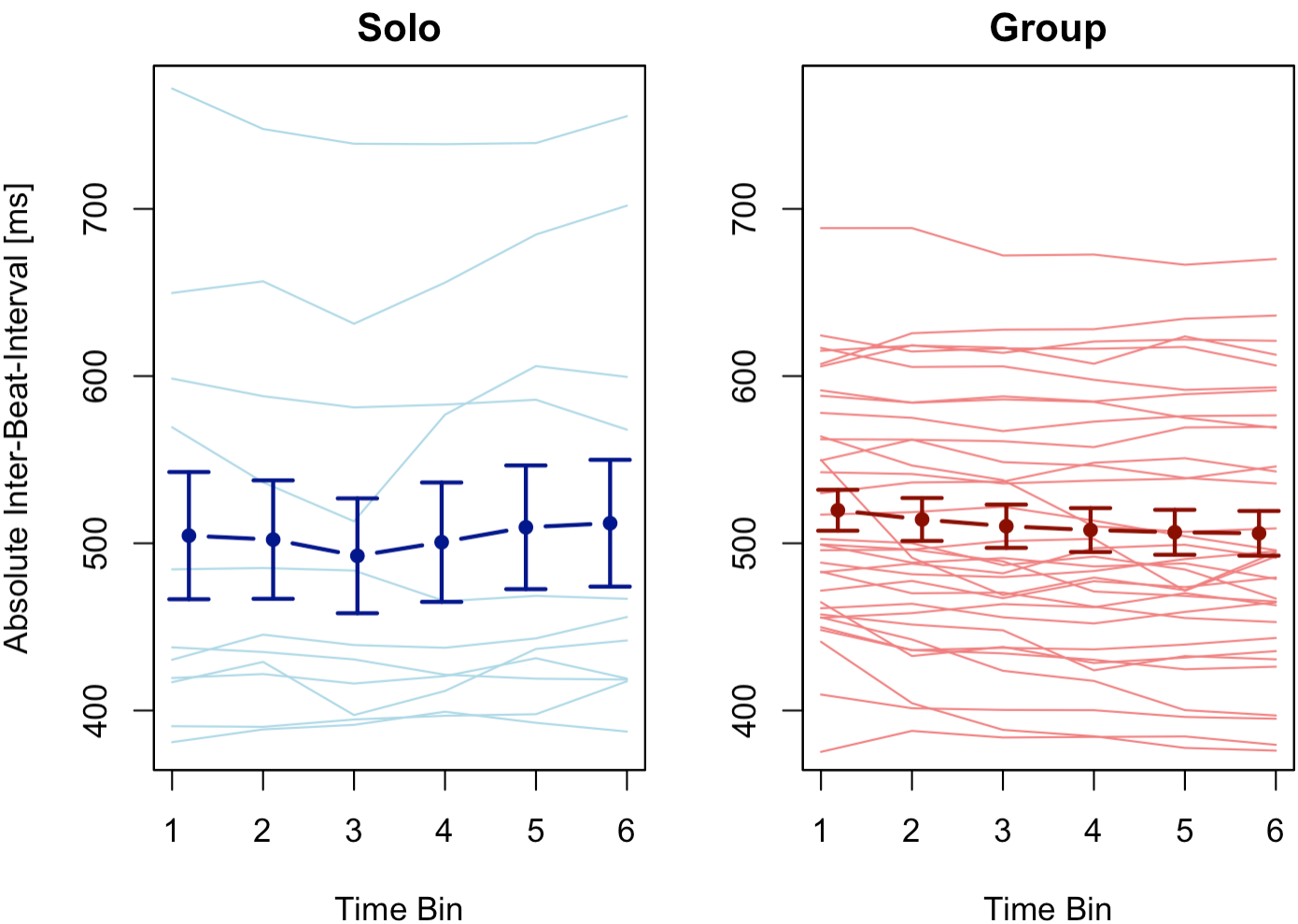
**

**SM3 – Small Groups vs. Large Groups in absolute Inter-Beat-Intervals**

We entered Absolute IBI in a 2 × 6 ANOVA with the between factor Group Type (Small Group or Large Group) and the within factor Time Bin (1 to 6).

Interaction*: *F*(5, 160) = 6.290, *p* = .002, η2 = 0.003

Main effect of Time Bin*: *F*(5, 160) = 9.635, *p* < .001, η2 = 0.005

Main effect of Group Type: *F*(1, 32) = 4.140, *p* = .050, η2 = 0.113

Post-hoc tests:

Differences between Small and Large Groups in

- Time Bin 1: *t*(29.097) = 1.504, *p* = .144
- Time Bin 2: *t*(29.991) = 1.760, *p* = .089
- Time Bin 3: *t*(29.921) = 2.058, *p* = .048
- Time Bin 4: *t*(29.790) = 2.208, *p* = .035
- Time Bin 5: *t*(30.479) = 2.298, *p* = .029
- Time Bin 6: *t*(29.761) = 2.262, *p* = .031


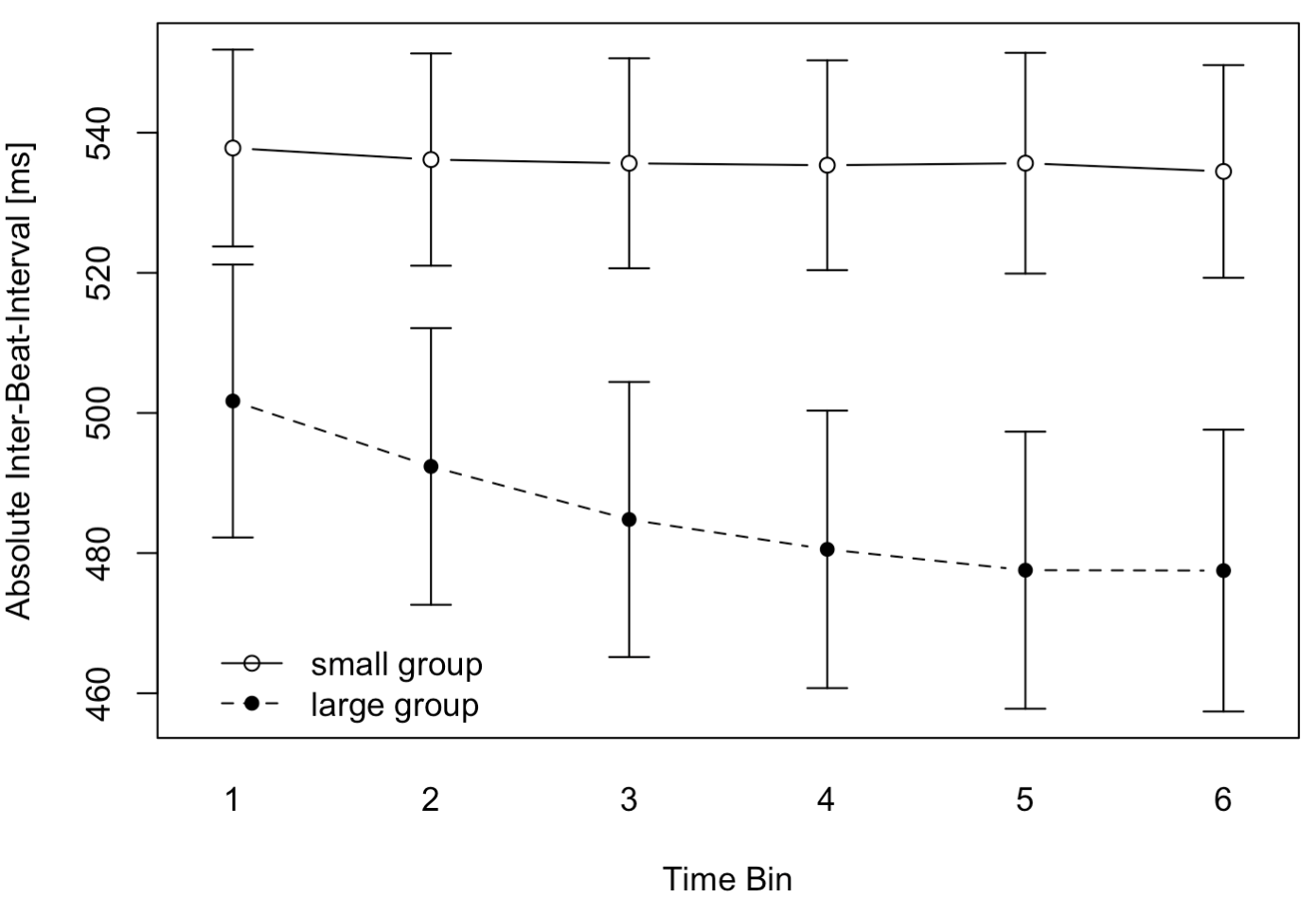


**SM4 Additional questions**

In addition to our main hypotheses, there were two further hypotheses included in the pre-registration which, in light of research conducted in the interim, we no longer consider to be well-motivated. The analyses pertaining to these two hypotheses are included here in the supplementary material. Please note that these two hypotheses concern two variables that could have served as additional predictors in a multiple regression. Removing them from the main text of this study is also the reason why we reverted to the calculation of simpler ANOVAs rather than the pre-registered multiple regressions.

**SM4.1 Movement similarity**

The first additional question is whether joint rushing is stronger in cases where interaction partners execute the same action than when they execute different actions. We reasoned that, if it is stronger when partners execute the same actions, this would point towards the involvement of mechanisms related to action mirroring [1]. However, the data of a lab study that was published in the meantime [2] provides evidence against such an involvement. Therefore, considering this new data and contrary to our pre-registration, we do not expect a significant difference in the current sample of videos either. Indeed, the results only showed a significant main effect of time bin.

We entered Absolute IBI in a 2 × 6 ANOVA with the between factor Movement Similarity (similar or not similar) and the within factor Time Bin (1 to 6).

Main effect of Time Bin*: *F*(5, 160) = 8.449, *p* < .001, η2 = 0.064

Main effect of Movement Similarity: *F*(1, 32) = 0.641, *p* = .429, η2 = 0.146

Interaction: *F*(5, 160) = 1.227, *p* = .299, η2 = 0.010


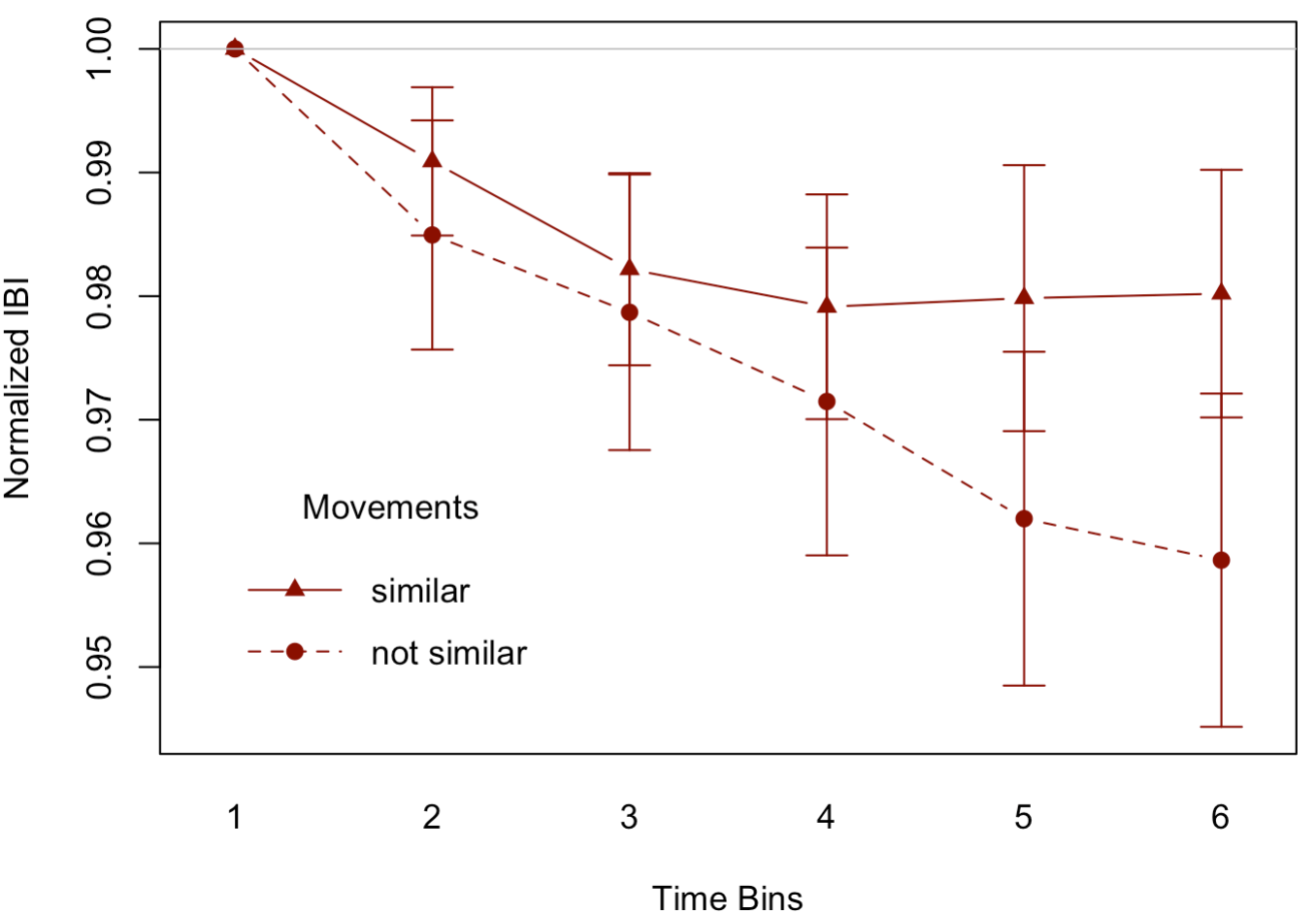


**SM4.2 Movement complexity**

The second additional question is about the effect of action complexity on joint rushing. If increased complexity resulted in more joint rushing, this would speak for the involvement of conscious control when trying to counteract joint rushing. Recently published data [3] that showed no significant differences in joint rushing between musicians and non-musicians, however, suggests otherwise. The task in this study should be less complex for coordination experts (i.e., musicians) than for non-experts (i.e., non-musicians). Therefore, we also did not expect a significant effect of Movement Complexity in this study. Indeed, the results only showed a significant main effect for time bin.

We entered Absolute IBI in a 3 × 6 ANOVA with the between factor Complexity (low, medium or high) and the within factor Time Bin (1 to 6).

Main effect of Time Bin*: *F*(5, 155) = 8.668, *p* < .001, η2 = 0.068

Main effect of Complexity: *F*(2, 31) = 1.396, *p* = .263, η2 = 0.062

Interaction: *F*(10, 155) = 1.544, *p* = .202, η2 = 0.025


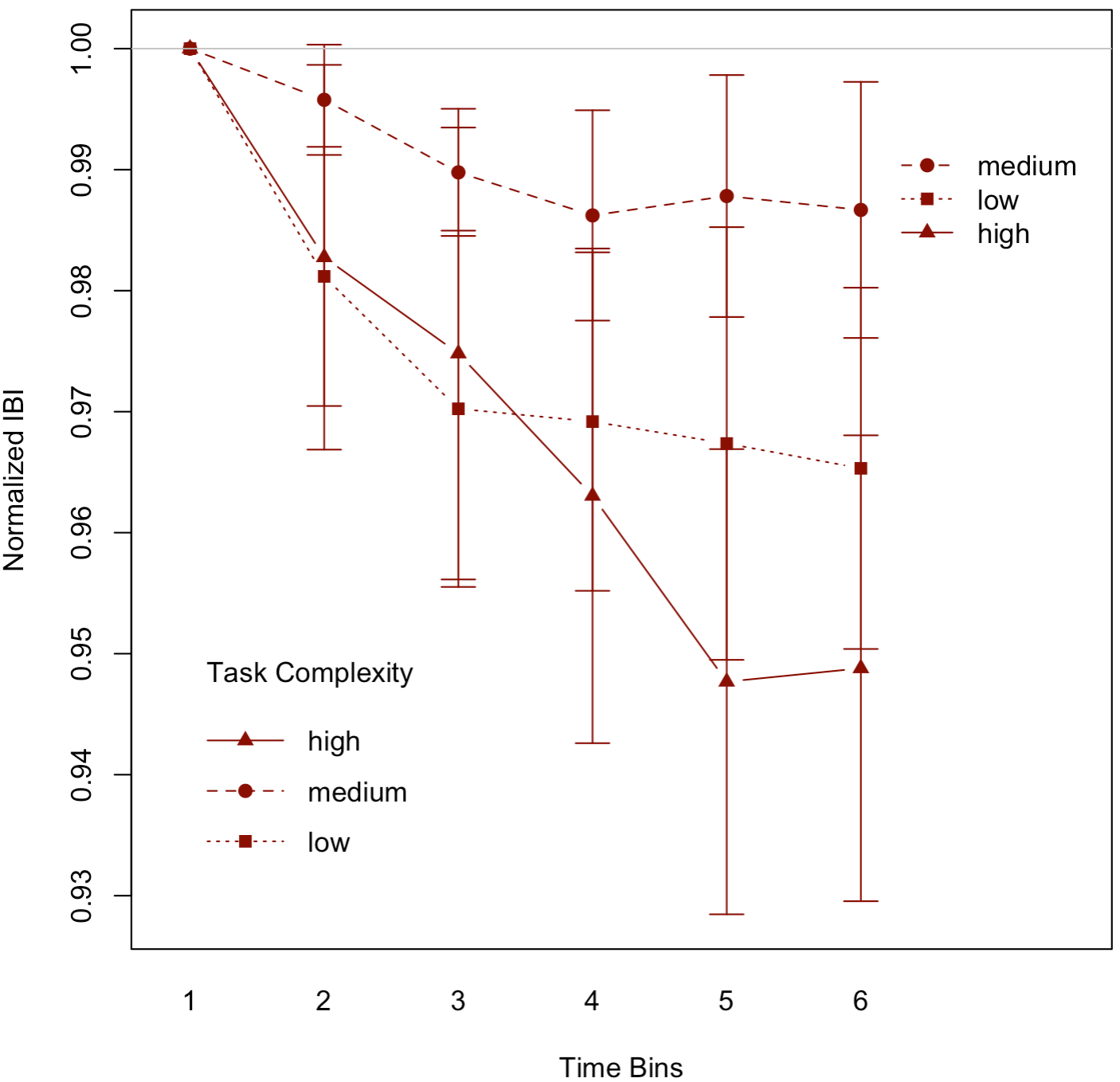


**SM5 ICCs for each video independently**

| **Video ID** | **ICC** | **p-value** |
| --- | --- | --- |
| -3MVWpXrB5o | 0.767 | 0.014 |
| -6VD_ZdTzPk | 0.972 | 0.000 |
| 0IOMI5GGP1g | 0.973 | 0.000 |
| 23Xust2zxbM | 0.854 | 0.004 |
| 3ikYFd9vTbk | 0.620 | 0.053 |
| 5AhrTeBVW4M | 0.986 | 0.000 |
| 5i8rG5rKzkY | 0.998 | 0.000 |
| 6XDQutQdHBI | 0.939 | 0.000 |
| 6izJ39ChKpI | 0.943 | 0.000 |
| 7TTAOuNToGs | 0.946 | 0.000 |
| B2UcIaIe2xE | 0.921 | 0.001 |
| E1SIF3REWBs | 0.917 | 0.001 |
| EULpbJQLzSg | 0.981 | 0.000 |
| EqusX23ZkAk | 0.883 | 0.002 |
| FWmoLP7A0MY | 0.952 | 0.000 |
| GP_aV_SdJLc | 0.952 | 0.000 |
| IwkPtUtNrvw | 0.923 | 0.001 |
| LQTol51C4oU | 0.994 | 0.000 |
| LmC6Qyjk8cw | 0.944 | 0.000 |
| P_POu_za5h0 | 0.564 | 0.076 |
| SX_pGYojtxc | 0.954 | 0.000 |
| SkHGhGpaW5k | 0.571 | 0.073 |
| U90mzAvCHGA | 0.978 | 0.000 |
| UY3XxnRtu48 | 0.629 | 0.050 |
| VVLl0C65NR8 | 0.886 | 0.002 |
| VZRypNsVBns | 0.988 | 0.000 |
| XBuJJ_luB90 | 0.480 | 0.118 |
| eIiYQN3pvt4 | 0.595 | 0.063 |
| f8bkvO1XpzM | 0.807 | 0.008 |
| fBeyDR84qKY | 0.982 | 0.000 |
| g7Wjl9x4N3U | 0.969 | 0.000 |
| hhHZ5tAruA4 | 0.976 | 0.000 |
| i0HEqE53WL4 | 0.946 | 0.000 |
| l0XdDKwFe3k | 0.982 | 0.000 |
| n0qs4_bY4vg | 0.983 | 0.000 |
| oFeWJaqqLrQ | 0.833 | 0.006 |
| pH6HYGjdc48 | 0.854 | 0.004 |
| pf_xLrhjjfI | 0.941 | 0.000 |
| r1ZQbEKiZ8o | 0.904 | 0.001 |
| u6cKxw0zkfc | 0.870 | 0.003 |
| u7yVMspnKTc | 0.575 | 0.071 |
| uhRAOFx-vLY | 0.635 | 0.048 |
| wYcLAC63pGI | 0.396 | 0.168 |
| wbxidzAdi4c | 0.664 | 0.038 |
| xISEWJrs4iU | 0.562 | 0.077 |

**SM - Literature**

1 Brass, M., Bekkering, H., & Prinz, W. (2001). Movement observation affects movement execution in a simple response task. *Acta Psychologica*, 20.

2 Wolf, T., Vesper, C., Sebanz, N., Keller, P. E., & Knoblich, G. (2019). Combining Phase Advancement and Period Correction Explains Rushing during Joint Rhythmic Activities. *Scientific Reports*, 9(1), 9350. <https://doi.org/10.1038/s41598-019-45601-5>

3 Wolf, T., & Knoblich, G. (2022). Joint rushing alters internal timekeeping in non-musicians and musicians. *Scientific Reports*, *12*(1), 1190. <https://doi.org/10.1038/s41598-022-05298-5>
